# Hyperglycemia acts in synergy with hypoxia to maintain the pro-inflammatory phenotype of macrophages

**DOI:** 10.1371/journal.pone.0220577

**Published:** 2019-08-15

**Authors:** Mangesh Morey, Peadar O'Gaora, Abhay Pandit, Christophe Hélary

**Affiliations:** 1 CÚRAM, Centre for Research in Medical Devices, National University of Ireland Galway, Galway, Ireland; 2 UCD School of Biomedical and Biomolecular Science, University College Dublin, Belfield, Dublin, Ireland; 3 Sorbonne Université, CNRS, Collège de France, Laboratoire de Chimie de la Matière Condensée de Paris,place Jussieu, Paris, France; National Institutes of Health, UNITED STATES

## Abstract

Diabetic foot ulcers (DFUs) are characterized by a chronic inflammation state which prevents cutaneous wound healing, and DFUs eventually lead to infection and leg amputation. Macrophages located in DFUs are locked in an pro-inflammatory phenotype. In this study, the effect of hyperglycemia and hypoxia on the macrophage phenotype was analyzed. For this purpose, a microarray was performed to study the gene expression profile of macrophages cultivated in a high glucose concentration. Hyperglycemia upregulated the expression of pro-inflammatory cytokines such as TNF-α, IL-1, IL-6, chemokines and downregulated the expression of two receptors involved in phagocytosis (CD 36 and Class B scavenger type I receptors). In addition, eleven anti-apoptotic factors were upregulated whereas three pro-apoptotic genes were downregulated. Subsequently, the contribution of hypoxia and hyperglycemia to chronic inflammation and their potential synergistic effect was evaluated on activated THP-1 derived macrophages. A long term post activation effect (17 hours) was only observed on the upregulation of pro-inflammatory cytokines when hypoxia was combined with a high glucose concentration. In contrast, hyperglycemia and hypoxia did not have any effect on wound healing molecules such as TGF-β1. Taken together, the results show that hyperglycemia acts in synergy with hypoxia to maintain a chronic inflammation state in macrophages.

## 1. Introduction

Diabetic foot ulcers are the most common, painful and crippling complications of diabetes mellitus [1]. These pathologies affect 3% of the diabetic population each year and this figure is expected to rise due to the increasing prevalence of diabetes [2]. In their lifetime, diabetic people have a 25% risk of developing a diabetic foot ulcer (DFU) [3]. Impaired wound healing is the main characteristic of DFU and can lead to infection and eventually to amputation. In the developed world, 85% of amputations are the consequence of DFU [4].The major underlying pathologies that cause DFU are peripheral neuropathy, vascular diseases and anatomic deformation [5]. It is well known that chronic hyperglycemia leads to oxidative stress and abnormal glycations of proteins which negatively impact nerve function. This neuropathy affects the motor and sensory components of the nervous system. In addition, persistent hyperglycemia leads to hypercoagulability of peripheral arteries which triggers thrombosis and ischemia in lower limbs [6]. As a result, foot deformation and ulceration can occur. Diabetic foot ulcers are chronic wounds characterized by chronic inflammation, massive extracellular matrix breakdown, impaired epithelialization and hypoxia [7]. The chronic inflammation is due to the presence of large numbers of inflammatory cells such as neutrophils and macrophages.

Macrophages play a key role in wound healing as they orchestrate the switch between the inflammation and proliferative phase [8]. After an injury, monocytes infiltrate the wound and differentiate into pro-inflammatory macrophages called M1 to clean the wound. At this stage, they secrete reactive oxygen species, inflammatory cytokines (IL-1, TNF-alpha, IL-6) and matrix metalloproteinases such as MMP-9 and MMP-2 [9]. Under the effect of cytokines from lymphocytes (IL-4 and IL-13), their phenotype evolves to become wound healing macrophages called M2. These cells promote granulation tissue formation and angiogenesis by the production of VEGF, TGF-**β**, PDGF and IGF-1 [9]. Another type called “pro-resolving macrophages” is dedicated to the resolution of inflammation via the secretion of IL-10 and TGF-**β**1 [2]. In pathological conditions, macrophages are locked in the M1 phenotype, thereby leading to chronic inflammation

Hypoxia in DFU creates conditions that are disadvantageous because the low oxygen tension induces the increased release of pro-inflammatory cytokines via the activation of NF-**κ**B signaling pathways [10, 11]. In addition, the switch from an oxidative to a glycolytic metabolism leads to increased production of ROS, cellular acidosis and high glucose uptake by cells via the increased gene expression of Glut-1 [12]. Nevertheless, hypoxia also has some positive effects in diabetic foot ulcers such as the promotion of angiogenesis via the activation of the HIF-1 signaling pathway [10].

A high glucose concentration in diabetic people could directly contribute to poor healing of wounds. The process of excessive glycation of proteins leading to AGEs (advanced glycation end products) increases the formation of ROS and the alteration of enzyme function [13]. It has been shown that the topical administration of glucose to non diabetic rats decreases the formation of granulation tissue and angiogenesis [14]. The accumulation of AGEs activates NF-**κ**B and induced TNF-α expression [15]. Some specific effects have been observed in high glucose concentration(s) including the inhibition of the proliferation of fibroblasts, keratinocytes and endothelial cells [13]. In addition, hyperglycemia reduces both the clearance of neutrophils and macrophages apoptosis whilst increasing secretion of MMPs [2, 16].

**Fig 1:**
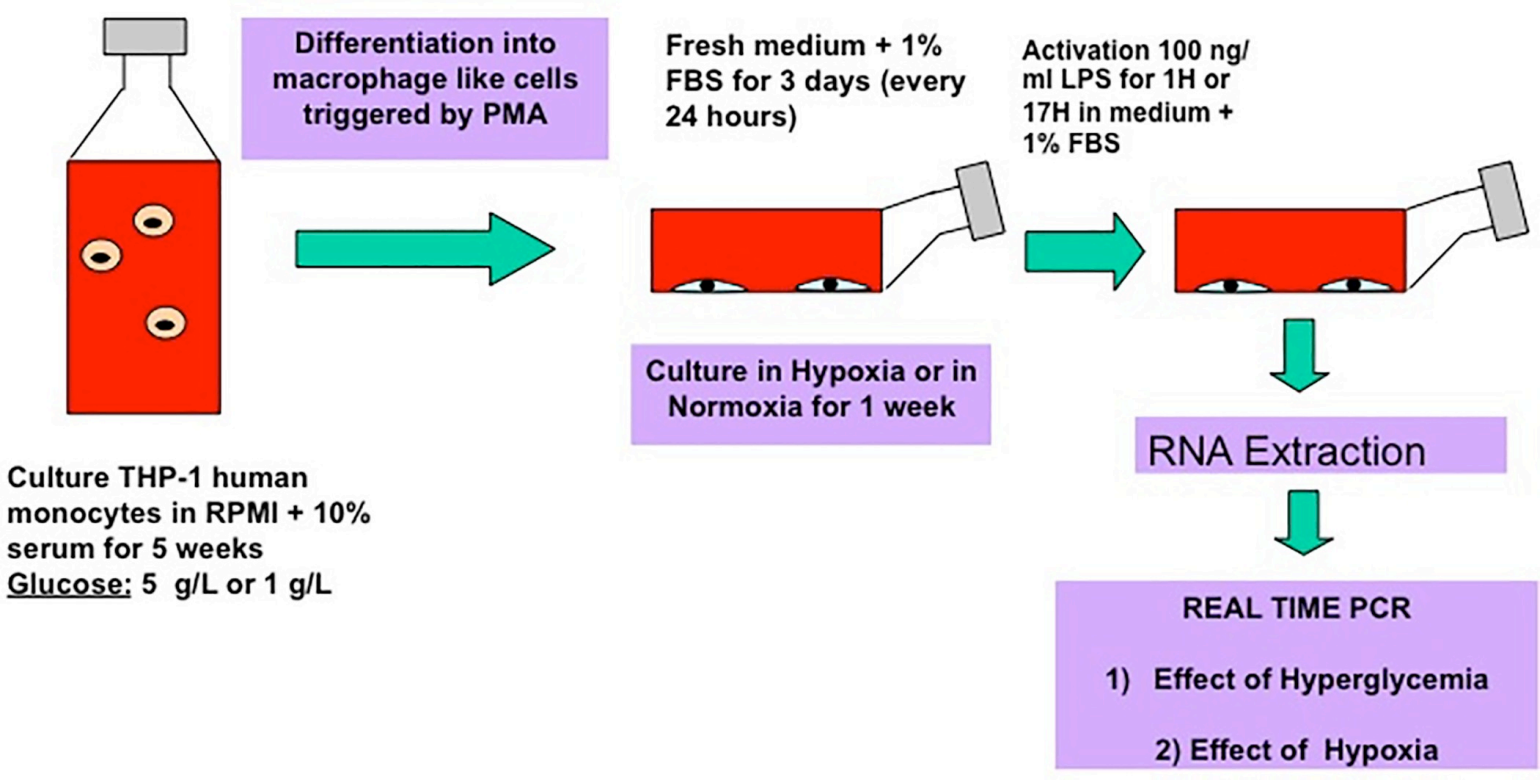
Differentiation and activation of macrophages cultivated in hyperglycemia and hypoxia.

In this study, we have analyzed the impact of hyperglycemia in combination with hypoxia on the gene expression profile of activated macrophages (Fig 1). Using microarray technology, we identified the genes that were upregulated or downregulated in activated macrophage derived THP-1 cells after 6 weeks culture in hyperglycemia and 4 days culture in hypoxia. Subsequently, the synergistic effect of high glucose concentration and a low oxygen pressure on the expression of relevant genes was studied in detail over 17 hours.

## 2 Materials and methods

### 2.1 Cell culture

The human myelogenous leukemia cell line THP-1 was obtained from ATCC. The cells were maintained in RPMI 1640 (GIBCO-BRL) supplemented with 10% fetal bovine serum (FBS), penicillin (100 U/ml), and streptomycin (100 μg/ml) at 37°C in a 5% CO_2_ humidified incubator. THP-1 cells were cultivated for 6 weeks either in hyperglycemia (4.5g/L glucose) or normoglycemia (1g/L glucose) before differentiation. During the cell culture, cell density was maintained between 2 and 8 x 10^5^ cells/ml.

### 2.2 Macrophage differentiation and activation

The mature macrophage-like state was induced by treating THP-1 cells for 24 hours with phorbol 12-myristate 13 acetate (PMA) at 100 ng/ml diluted in serum free medium (Fig 1). 500,000 cells/well were seeded into a 24 well plastic well plate. The following day, plastic-adherent cells were washed twice with cold, sterile Dulbecco’s phosphate-buffered saline (PBS) and incubated with fresh RPMI-1640 lacking PMA, but containing 1% FBS, penicillin (100 U/ml) and streptomycin (100 μg/ml). Macrophage-like cells, maintained in hyper or normoglycemia, were then placed in a hypoxic chamber (1% O_2_) for three days to mimic the chronic wound environment. On the fourth day, cells were activated with 100 ng/ml Lipopolysaccharide (LPS) in RPMI-1640 medium containing 1% FBS and collected after 1 and 17 hours.

### 2.3 RNA extraction and TNF-alpha gene expression

Total RNAs were extracted from macrophages after 1 hour or 17 hours post LPS activation using a variant of Trizol isolation. Briefly, TriReagent (Invitrogen) was added onto the cells, then lysates were collected into 1·5 ml tubes. Phase separation was performed by adding chloroform (Sigma-Aldrich), and total RNA was purified using an RNeasy kit (Qiagen), according to the supplier’s recommended procedure. Total RNA quantity and purity were determined using an ultraviolet spectrometer (NanoDrop ND-1000 Spectrophotometer, NanoDrop Technologies). RNA integrity was checked electrophoretically using the RNA 6000 Nano LabChip kit on Agilent Bioanalyser 2100 (Agilent Technologies).

Reverse transcription (RT) was performed on samples collected after an LPS activation for one hour using the ImProm-II RT system according to the manufacturer’s protocol (Promega). To control the macrophage activation by LPS, gene transcription of Tumor Necrosis Factor Alpha (TNF-Alpha) was examined using real-time RT-polymerase chain reaction (PCR). Reactions were performed and monitored using an ABI *StepOnePlus* Real-Time PCR System (Applied Biosystems) using the Sybr Green chemistry (Qiagen) and specific primer sequence of TNF-Alpha (NM_000594.2). Gene transcription was inferred from calibration samples (Usually Control samples) and normalized in relation to transcription of the housekeeping gene GAPDH (The list of primers is included as S1 Table). The absolute quantification method using an arbitrary standard curve was used to calculate relative gene expression. Ratio were calculated by using no activated macrophages cultured with 1g/L glucose in normoxia as calibrator point.

### 2.4 Microarray

Microarrays were performed on six biological replicates using GeneChip Human Genome U133 Plus 2·0 Array (Affymetrix) at University College Dublin. The comparison in terms of gene expression was carried out between activated macrophages cultivated in hyperglycemia and those cultivated in normoglycemia. For this experiment, all macrophages were cultivated in hypoxia. Briefly, 2 μg of total RNA was converted to cRNA and cleaned up. The Biotin labeled cRNA was then synthesized. Unincorporated NTPs were removed and quality was assessed using an Agilent 2100 bioanalyzer. 100ng of total RNA was used for cDNA synthesis for generating unlabelled cRNA. This cRNA was then cleaned up and reverse transcribed in the second cycle first strand cDNA synthesis, double-stranded cDNA cleaned up, amplified and labeled. The newly synthesized biotin labeled cRNA was cleaned removing unincorporated NTPs and quality assessed. 25μg of cRNA generated in the *in vitro* transcription (IVT) reaction was fragmented using 5X fragmentation buffer and RNase-free water. The fragmentation reaction was carried out at 94°C for 35 mins to generate 35–200 base fragments for hybridization. Quality was assessed at this stage. Prior to hybridization, the adjusted cRNA yield was calculated for total RNA carryover in the IVT reaction. 15μg of fragmented cRNA made up into a hybridization cocktail was added to HG-U133 Plus 2.0 array and hybridized for 16hrs at 45°C, washed and stained on a fluidics station. Once completed, the samples were scanned using GeneChip Scanner 3000. The results were obtained as CEL files. The raw microarray signal intensities were read into R and pre-processed using the affy and GCRMA packages of the BioConductor project [17]. Gene expression was analyzed using Entrez Gene probe set remappings from MBNI [18]. Differentially expressed genes (DEGs) were identified using linear modeling and a modified t-test implemented in the Limma package [19]. All *p* values were adjusted for multiple testing using the false discovery rate method [20]. Gene filtering on normalized intensity followed by fold changes >2-fold and P-Value of < 0·05 was used to generate a list of genes for expression profiles. Annotation provided by Affymetrix was extended using DAVID. Functional clustering of lists of differentially expressed genes was generated by comparing them against the ontology terms for molecular function, cellular composition and biological processes using Gene Ontology databases (GO), the Medical Subject Heading Terms Database) (MeSH) and KEGG PATHWAY Database.

### 2.5 Real time PCR

For validating the microarray data and to assess the effect of glycemia and hypoxia on gene expression over a 17 hour activation period by LPS, real time PCR was performed. The isolated RNA was first reverse transcribed using ImProm-II Reverse Transcription System (Promega). The cDNA thus obtained was then used for real time PCR reaction (ABI *StepOnePlus* Real-Time PCR System, software v2·1) with specifically designed primers (S1 Table) and Fast SYBR Green Master Mix (Applied Biosystems).

Gene expressions were quantified using the absolute quantification method (n = 6). Cycling conditions were: initial denaturation at 94°C for 5 min, followed by 40 cycles consisting of a 10 s denaturation at 94°C, a 15 s annealing at 59°C and a 15 s elongation at 72°C. Then, a melting curve was obtained for each sample by increasing the temperature from 59°C to 97°C at a rate of 0·11°C/s.

The results were analyzed using the absolute quantification with arbitrary values. For this purpose, a standard curve was carried out for each target and reference gene. Primer efficiencies were calculated in each experiment from the standard curve carried out in the same plate as the quantified samples. For each sample, a ratio target gene/reference gene was calculated and compared with a calibrator point. This calibrator point was the cDNA obtained from the control samples (No activated macrophages cultured in normoxia and normoglycemia). The value 1 was given to the mean of ratios for the control samples (n = 6). Arbitrary values were then calculated for each condition by comparison with the value 1, using the ratios.

### 2.6 Statistical analysis

Results are presented as mean ± SD (standard deviation). Statistical significance was assessed using one way analysis of variance (ANOVA) followed by Mann Witney (compare all pairs of groups) posthoc test. The level of significance in all statistical analyses was set at a probability of P < 0·05. Prism (Excel Stat) software was used for all data analysis.

## 3. Results

### 3.1 Gene expression profile of macrophages cultivated in hyperglycemia and hypoxia

**Fig 2:**
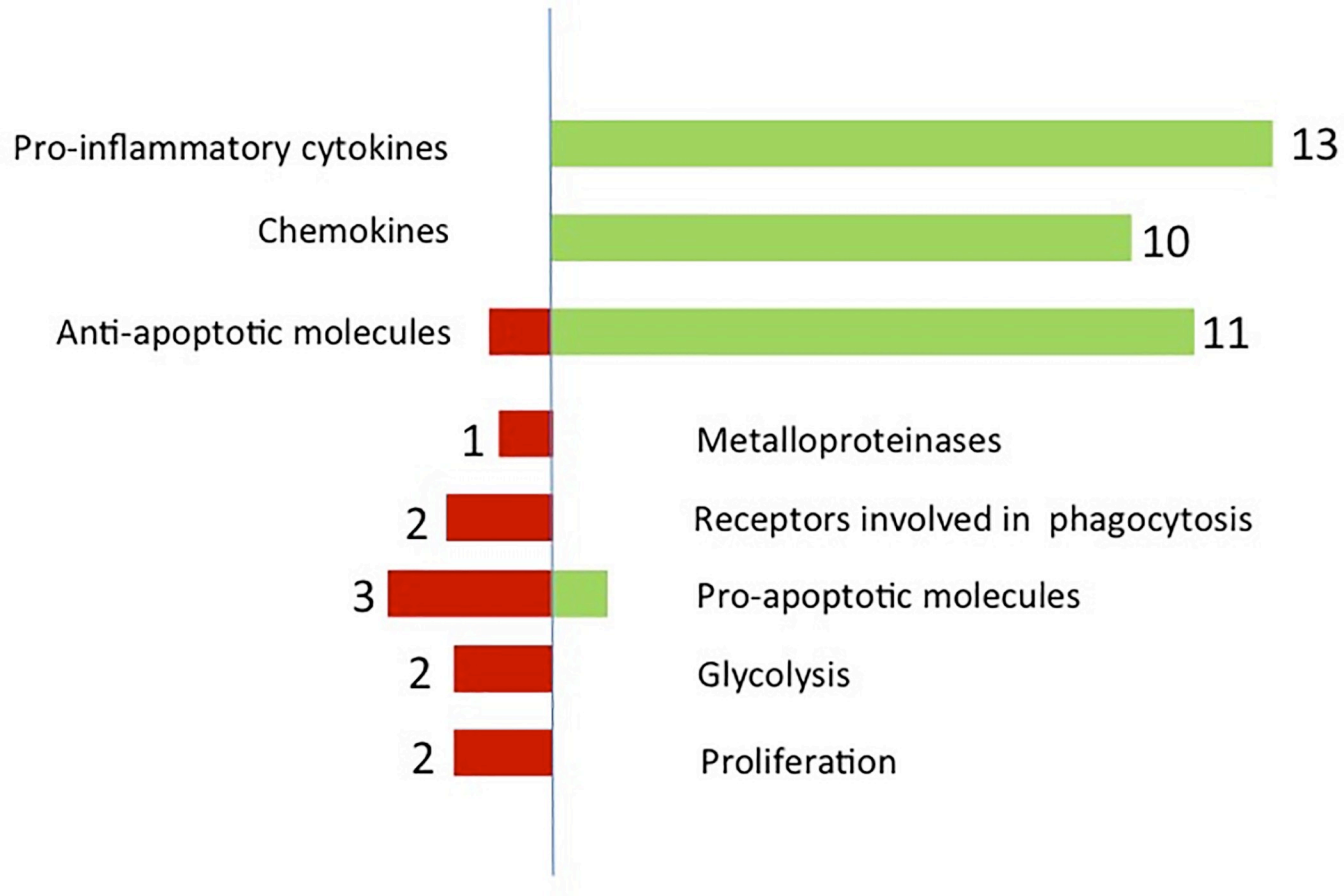
Epigenetic changes in hyperglycemic macrophages. Overview of genes upregulated and dowregulated in a high glucose environment and hypoxia.

The microarray revealed that 546 genes were statistically up or downregulated in hyperglycemia. Filtering for gene expression modulated at least 2 fold, only 54 genes were found to be upregulated and 94 downregulated in hyperglycemia. Among these genes, thirteen proinflammatory cytokines and ten chemokines were found to be upregulated (Fig 2). In contrast, TGF-β1, a crucial cytokine promoting wound healing was downregulated in hyperglycemia (2·01 fold lower). Moreover, CD36 and Scavenger receptor B, two genes involved in the process of phagocytosis, were also downregulated (Table 1). After analysis, the microarray revealed that three major signalling pathways were modulated in hyperglycemia. These pathways were the EGF (Epidermal Growth Factor) and Wnt5A (Wingless-type MMTV integration site family, member 5A) and NOD signalling pathways (Table 1). Eleven genes encoding for proteins with an anti-apoptotic effect were also upregulated and three pro-apoptotic genes were downregulated (Fig 2). Surprisingly, no genes encoding for metalloproteinases or other proteases were upregulated when cells were cultivated in hyperglycemia. Finally, only a few genes involved in glycolysis were modulated by the high glucose concentration. Only two were slightly downregulated for glycolysis and two for proliferation.

**Table 1:** Gene expression profile of THP-1 derived macrophages cultivated in hyperglycemia and hypoxia.

Symbol		Entrez Gene Name	Fold Change
**TNF-α**	Pro-inflammatory cytokines	Tumour necrosis factor alpha	3.50
**IL-1A**	Pro-inflammatory cytokines	interleukin 1 alpha	6.82
**IL-1B**	Pro-inflammatory cytokines	interleukin 1 beta	2.30
**IL-6**	Pro-inflammatory cytokines	interleukin 6	6.49
**CSF2**	Pro-inflammatory cytokines	colony stimulating factor 2 (GM-CSF)	6.49
**IL-24**	Pro-inflammatory cytokines	interleukin 24	4.00
**LIF**	Pro-inflammatory cytokines	interleukin 6 family cytokine	4.32
**CXCL1**	Chemokines	C-X-C motif chemokine ligand 1	3.73
**CXCL2**	Chemokines	C-X-C motif chemokine ligand 2	2.56
**CXCL3**	Chemokines	C-X-C motif chemokine ligand 3	1.94
**CXCL4**	Chemokines	C-X-C motif chemokine ligand 4	1.98
**CXCL5**	Chemokines	C-X-C motif chemokine ligand 5	4.56
**CCL4**	Chemokines	C-C motif chemokine ligand 4	2.68
**CCL19**	Chemokines	C-C motif chemokine ligand 19	2.17
**TGF-β1**	Wound healing cytokine	Transforming growth factor beta 1	-2.01
**CD-36**	Phagocytosis	CD36 molecule	-1.96
**SCARB-1**	Phagocytosis	Scavenger receptor class B member 1	-2.47

### 3.2. Effect of hyperglycemia and hypoxia on gene expression of inflammatory cytokines

**Fig 3:**
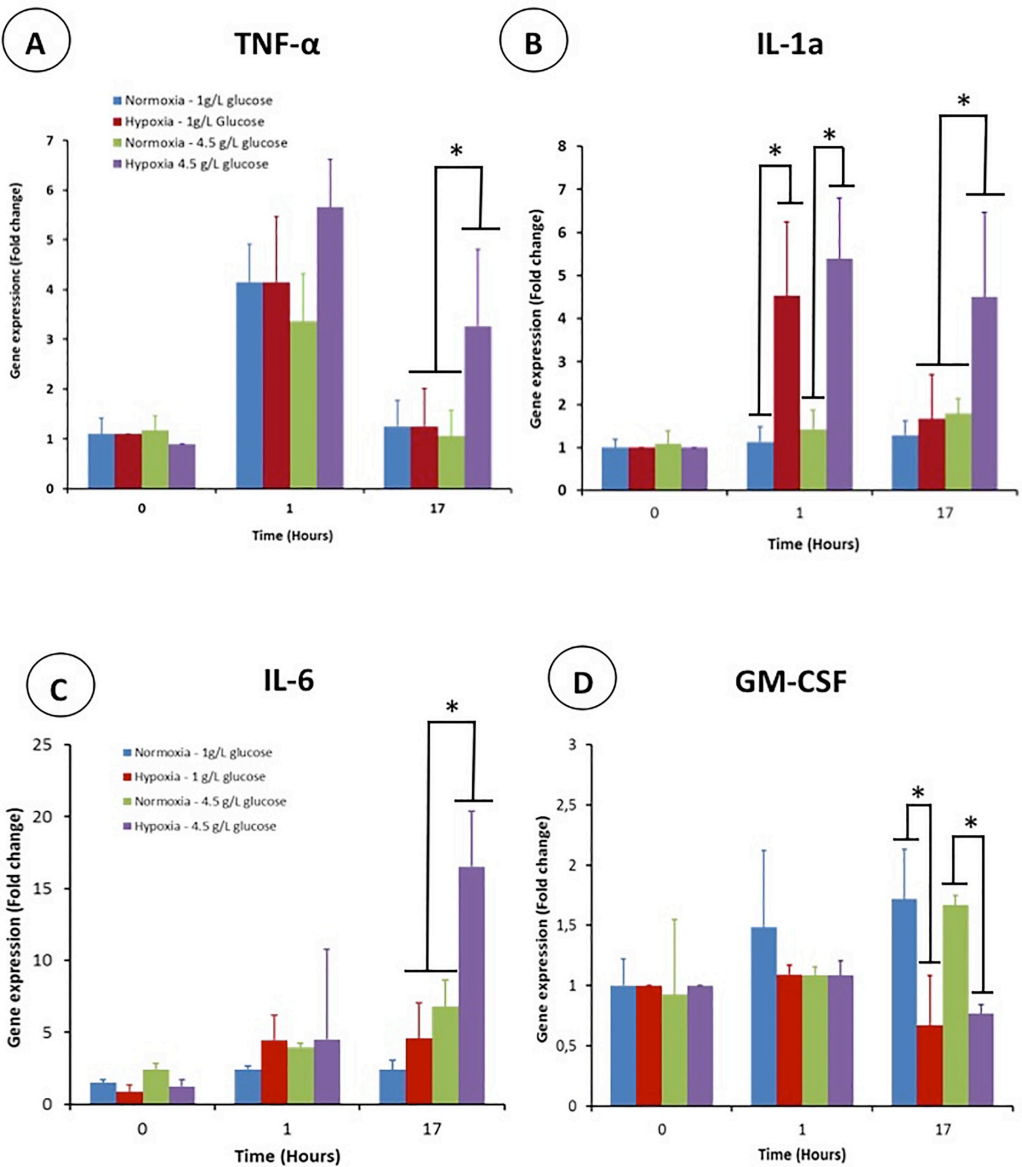
Gene expression profile of proinflammatory cytokines in activated THP-1 derived macrophages cultivated in hypoxia and/or hyperglycemia one hour and 17 after their activation by lipopolysaccharide (LPS). (A) TNF-α, (B) IL-1a, (C) IL-6, (D) CSF2, GM-CSF.

The impact of hypoxia and hyperglycemia on the gene expression of TNF- α, IL-1a, IL-6 and GM-CSF was analyzed in detail and compared to the results obtained with the microarray. Gene expression was measured 1 hour and 17 hours after macrophage activation by LPS. This activation triggered an increase of the TNF-α gene expression after one hour regardless of the culture conditions. The gene expression was around four times the basal level but was not significantly different whether the cells were cultivated in hypoxia or in hyperglycemia (Fig 3A). After 17 hours post activation, TNF-α gene expression recovered its basal level except for the condition hyperglycemia/hypoxia (three times the basal level). As a result, the combination of hypoxia and hyperglycemia has a synergistic effect for long term TNF-α gene expression.

Unlike TNF-α, the LPS activation did not increase the IL-1 gene expression after one hour when cells were cultivated in normoxia. In sharp contrast, hypoxia had a huge effect as the IL-1a gene expression was circa five fold higher for cells cultivated in hyperglycemia and normoglycemia. After 17 hours, the IL-1a expression decreased to its basal level in normoglycemia whereas that measured in hyperglycemic conditions remained high (Fig 3B). After 17 hours post-activation, hyperglycemia and hypoxia are required to maintain a high expression of IL-1A.

The addition of lipopolysaccharide to macrophages triggered a slight increase of IL-6 gene expression irrespective of the culture condition (Fig 3C). One hour post activation, a slight increase was observed in the normoxia/normoglycemia group (1·5–2 times the basal level). When hypoxia was combined with hyperglycemic conditions, IL-6 gene expression increased dramatically for this group after 17 hours post activation. Interestingly, cells cultivated in normoxia and hyperglycemia exhibited an increased expression of IL-6 after 17 hours compared to that after 1 hour (Fig 3C). In this case, hyperglycemia has an effect on its own but it was amplified by hypoxia.

Macrophage activation did not have any effect on GM-CSF gene expression of each group one hour after LPS addition (Fig 3D). A long term effect of hypoxia was observed as the gene expression of cells cultivated in hypoxia was half that of those cultivated in normoxia.

Therefore, hyperglycemia and hypoxia have a negative effect on inflammation as they upregulate the gene expression of the inflammatory cytokines TNF-α, IL-6 and IL-1 in activated macrophages.

### 3.3 Gene expression of receptors involved in phagocytosis

**Fig 4:**
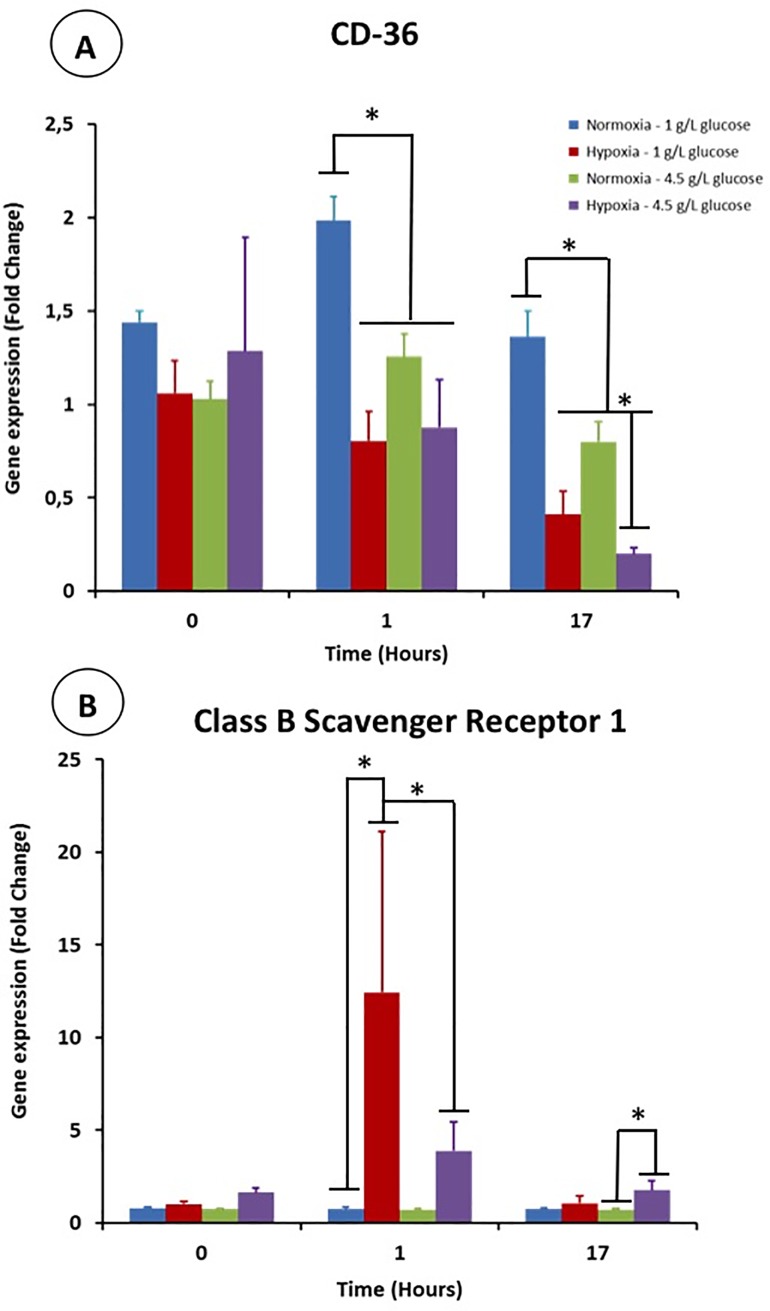
Gene expression of CD36 (A) and Class B Scavenger Receptor (B) in activated THP-1 derived macrophages cultivated in high glucose concentration and/or low O_2_ tension one hour and 17 hours after their activation by LPS.

The LPS activation of macrophages led to a slight increase of the CD-36 gene expression in normoxia and normoglycemia (Fig 4A). In contrast, this gene expression was not modulated when cells were cultured in hypoxia/hyperglycemia. Hypoxia and hyperglycemia had a long term effect on this gene expression. Low oxygen tension and high glucose concentration negatively impacted the CD-36 expression on their own. The gene expression measured after 17 hours post-activation was circa 50% and 75% lower in hyperglycemia and hypoxia, respectively. It is worth noticing that a synergistic effect of hyperglycemia and hypoxia was observed as the CD-36 expression was inhibited down to circa 90% for this condition at this time point.

Macrophages exhibited a drastic upregulation of the Class B Scavenger receptor gene when the cells were cultivated in hypoxia. No effect was observed in normal O_2_ condition (Fig 4B). This activation was partially inhibited in hyperglycemic condition because the CD-36 expression is 1/3 of that observed in normoglycemia (Fig 4B). After 17 hours post-activation, Class B Scavenger gene expression recovered its basal level irrespective of the culture conditions. However hypoxia had a slight positive effect when cells were cultured in hyperglycemia.

Hence, hypoxia and hyperglycemia decreases the abilities of activated macrophages for phagocytosis because the expression of CD-36 and Class B scavenger are downregulated.

### 3.4 Gene expression of cytokines involved in wound healing

**Fig 5:**
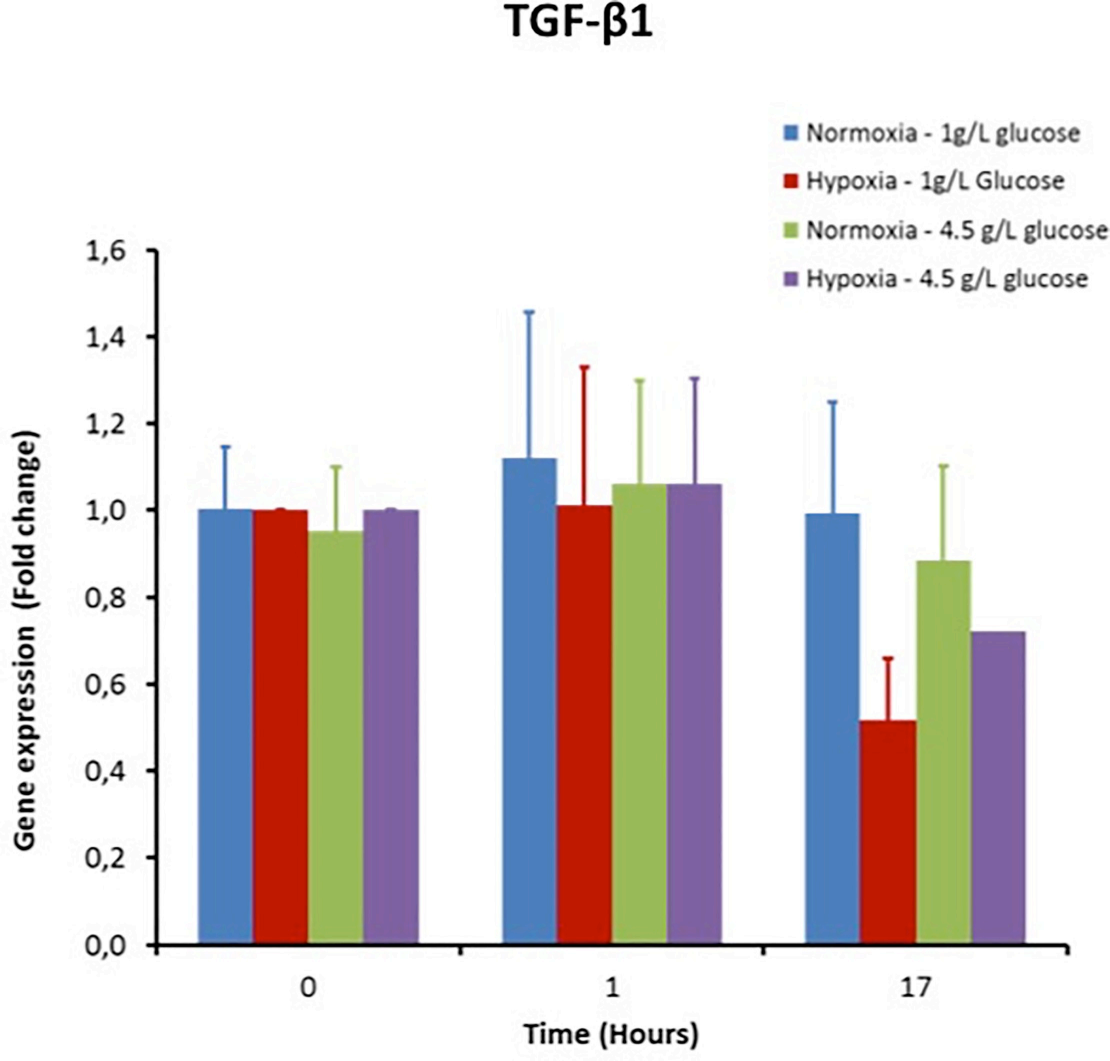
Impact of hyperglycemia and hypoxia in activated macrophages on the gene expression of TGF-β, the major wound healing molecule.

The TGF-β1 gene expression was not modified one hour after activation of macrophages regardless of the O_2_ and glucose conditions (Fig 5). This expression did not change after 17 hours post activation either. Hence a low O_2_ tension and hyperglycemic conditions does not have any impact on TGF-β production (Fig 5).

### 3.5 Gene expression of SOCS-3

**Fig 6:**
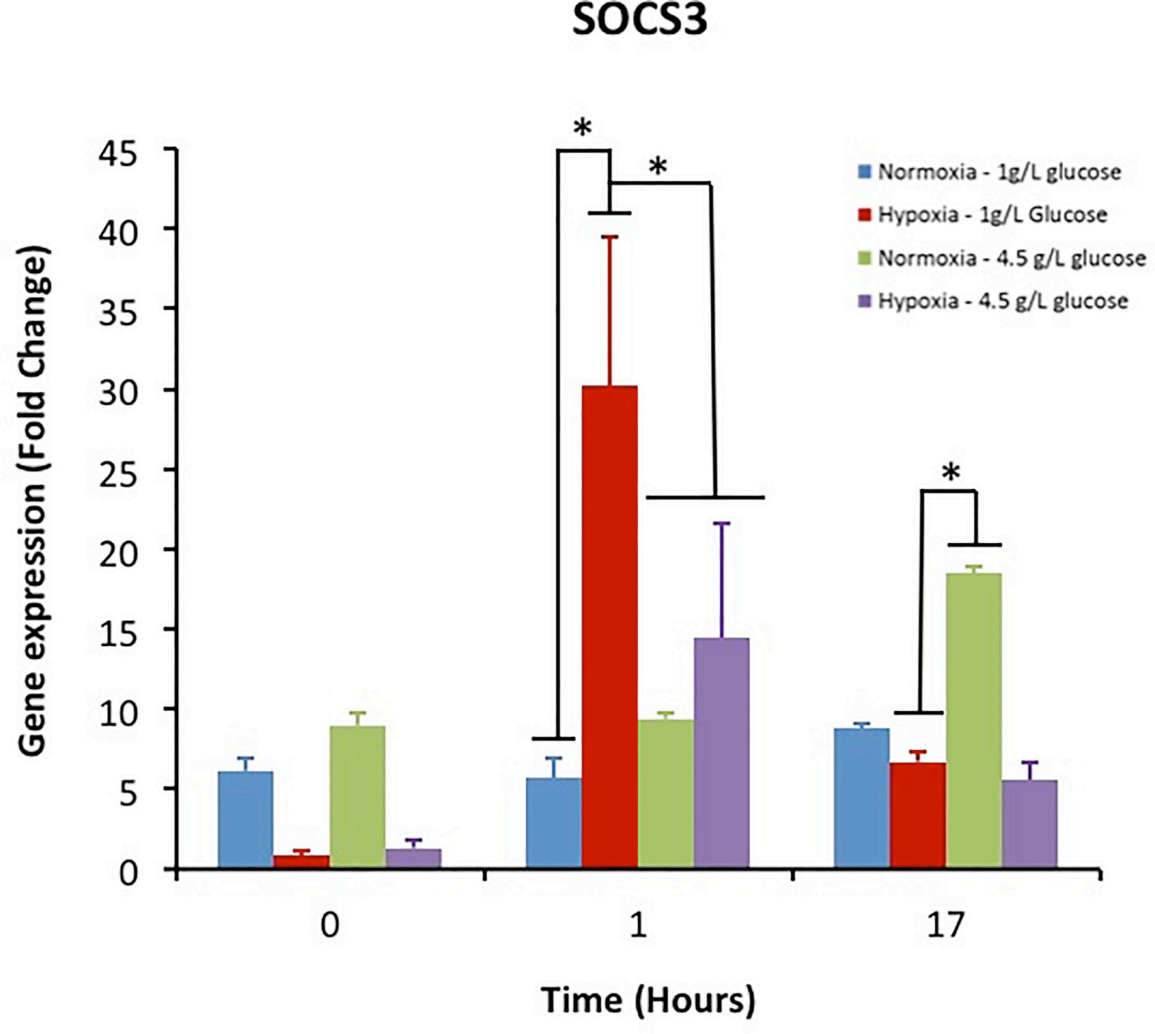
Impact of hyperglycemia and hypoxia on the gene expression of SOCS-3 in activated macrophages.

SOCS-3 was upregulated one hour after LPS activation when the cells were cultivated in hypoxia. This upregulation was higher for macrophages cultivated in normoglycemia (Fig 6). The SOC-3 gene expression decreased to its basal level after 17 hours in these groups. In contrast, the cells cultivated in normoxia and hyperglycemia exhibited an upregulation of SOCS-3.

## 4 Discussion

The goal of this study was to analyze the impact of hyperglycemia on the macrophage phenotype focusing on proteins involved in inflammation, proliferation, apoptosis, ECM breakdown and wound healing. For this purpose, a gene expression microarray analysis was performed on activated macrophages cultured in a hyperglycemic and hypoxic environment with a low quantity of bovine serum with the aim of mimicking the chronic wound milieu. Subsequently, the effect of hyperglycemia and hypoxia were analyzed separately to understand their contribution in the chronic wounds. Lastly a potential synergistic effect of high glucose concentration and low O_2_ tension was evaluated.

Hyperglycemia has several detrimental effects on human homeostasis. A chronic high glucose concentration leads to a process of protein glycation and the production of advanced glycation endproducts (AGEs). [13, 21]. AGEs promote macrophage activation via NF-**κ**B and stimulate the production of reactive oxygen species (ROS) [22]. As a consequence, diabetes predisposes to epigenetic changes which lead to chronic inflammation [23]. The microarray results show that 13 pro-inflammatory cytokines and 10 chemokines were upregulated in hyperglycemia, thereby confirming the perpetual dysregulation of the inflammatory homeostasis. Pro-inflammatory macrophages are more metabolically active in hyperglycemic conditions and exclusively use glucose as a source of energy [24]. Hence, this mode of energy production can contribute to the failure to resolve inflammation.

Chronic wounds are characterized by the recruitment and the persistence of immune cells in the wound bed (neutrophils and macrophages) [25]. The results showed the upregulation of 11 anti-apoptotic genes and the downregulation of 3 pro-apoptotic genes, indicating the direct impact of hyperglycemia on the large number of macrophages inside the cutaneous wound bed. One major feature of impaired wound healing is the massive breakdown of extracellular matrix. High glucose concentration triggers the production and secretion of metalloproteinases such as MMP-9 and MMP-2 by fibroblasts, keratinocytes and macrophages [25, 26]. In our conditions, hyperglycemia did not have a direct effect on proteases as only MMP-7 was affected. In addition, this enzyme was slightly downregulated.

Lipopolysacharide (LPS) is an outer membrane component of Gram negative bacteria which activates macrophages [27]. LPS contact with TLR receptors orientates macrophages towards a pro-inflammatory M1 phenotype. This phenotype is characterized by the production of inflammatory cytokines such as IL-6, IL-1, TNF-α, reactive species of oxygen (ROS) and NO [28]. The expression of inflammatory cytokines is based on the NF-**κ**B activation in macrophages [29]. AGEs interacting with RAGE, their membrane receptor, can be a continuous activator) of NF-**κ**B. As a result, AGEs increase the production of pro-inflammatory cytokines as previously described [30].

Hypoxia is associated with the activation of hypoxia inducible factors (HIFs) which is the key mediator of the induction of IL-6, IL-1, TNF-α [31]. Hence, hypoxia and hyperglycemia could have a synergistic effect on the production of pro-inflammatory cytokines. In addition, a cross-talk exists between HIF and NF-**κ**B to increase this production. We analyzed in detail the impact of hypoxia and high glucose on cytokine production with a kinetic view. After one hour post LPS activation, the combination of hypoxia and hyperglycemia had a dramatic effect on the expression of TNF-α and IL-6. The combination of hyperglycemia and hypoxia is required to induce a sustained production of pro-inflammatory cytokines as the same phenomenon was observed for TNF-α and IL-6. Beside its major role in inflammation, it has been recently shown that IL-6 could have anti-inflammatory effects via modulation of macrophage phenotype [32]. IL-6 promote the M2 phenotype of macrophages by inducing the expression of the IL-4 receptor [32]. In this study, the IL-4 receptor was not upregulated. Several studies have reported on the anti-inflammatory effect of IL-6 and the dependency on the concentration. In this study, Il-6 was dramatically upregulated and orientated its action towards chronic inflammation [32]. Regarding IL-1, only hypoxia had a short term impact on the expression of this cytokine. An effect was observable 17 hours post activation for the cells cultivated in hypoxia and hyperglycemia. This shows their importance for a long term effect on inflammation. Moreover, the sustained and prolonged production of IL-1 contributes to diminish wound healing by activating TLR receptors and maintaining macrophages in a M1 phenotype [33].

Granulocyte macrophage colony-stimulating factor (GM-CSF) is highly upregulated in hyperglycemic conditions. GM-CSF is produced during the inflammation phase and is a marker of M1 macrophages [34]. This cytokine stimulates the production of chemokines such as CCL2 and CCL3 and is involved in the recruitment of myeloid cells within the wound [33]. The GM-CSF expression is induced by pro-inflammatory cytokines such as IL-1 and TNF-alpha. As a consequence, the high production of pro-inflammatory cytokines by high glucose and low O2 tension increases the expression of GM-CSF, which has also a negative effect on inflammation. In our conditions, GM-CSF was not impacted by hyperglycemia which is not consistent with the results of the micro array.

Suppressor of cytokine signaling 3 (SOCS3) is associated with the pro-inflammatory M1 phenotype of macrophages. In addition, SOCS3 decreases the phagocytic activities of macrophages for apoptotic neutrophils. The decrease of clearance of dead neutrophils impedes the resolution of inflammation and a pro-inflammatory environment shows a strong upregulation of SOCS3 [35, 36]. Hyperglycemia seems to have a short term negative effect on SOCS3. Surprisingly, hyperglycemia seems to favour the resolution of inflammation at this time point. However, hyperglycemia has a negative effect after 17 hours when the cells are cultivated in hyperglycemia. As SOCS-3 is upregulated in this study, this confirms the inflammatory effect of IL-6 in hyperglycemia. It has been shown this cytokine has an anti-inflammatory effect only when SOCS-3 was downregulated or ablated [37].

Hyperglycemia combined with hypoxia also led to the upregulation of a panel of chemokines. Among them, CCL-4 is of great interest because it activates neutrophils which can trigger neutrophilic inflammation [38, 39]. In addition, this chemokine triggers the production of pro-inflammatory cytokines. Five C-X-C chemokines (CXCL 1- CXCL5) were also upregulated in hyperglycemia. For example, CXCL2 is highly expressed. This chemokine is expressed in response to TNF-alpha via NF−κ_B_ activation and triggers the expression of IL-1, IL-6 and iNOS [40]. Moreover, CXCL2 recruit neutrophils to infection sites. Overall, the other chemokines have the same effect, recruiting leucocytes in the wound. Hence, hyperglycemia and hypoxia create a vicious circle which maintains a high inflammation in the wound and prevents the switch from the inflammatory phase to the proliferative one.

Phagocytosis of dead cells is required for the resolution of inflammation and the transition towards the proliferative phase [41] because impaired cell clearance has been observed in diabetic wounds [42]. CD36 is a member of the class B scavenger receptor family found in macrophages. CD36 is an efferocytosis receptor which acts in combination with α_v_β_3_ integrin to engulf dead neutrophils [41]. Unlike the normoglycemic conditions, CD36 expression does not increase in hyperglycemia one hour after LPS activation. This result shows the impaired phagocytic activities of macrophages cultivated in high glucose. In addition, CD36 mediate(s) the bacteria phagocytosis and the production of inflammatory molecules such as IL-8 [43]. Hence, the absence of an upregulation of CD36 following the activation by LPS suggests the lower ability of macrophages to combat infection when they are in a hyperglycemic milieu.

Class B scavenger type I receptors (CLA-1) are also involved in the pathogens recognition and the removal of apoptotic cells. They have a lot of structural similarities with CD36 [43]. They also have an effect on cytokine production as Knock Out CLA-1 mice expressed more inflammatory cytokines than the wild type [43]. The results showed that hypoxia is an important stimulus for Class B scavenger expression because its expression is multiplied by 12 in hypoxia over that in the normoxic conditions. Hyperglycemia negatively modulates this upregulation showing once again the impaired phagocytic abilities of diabetic macrophages, thereby settling down the chronic inflammation in the cutaneous wound.

TGF-B1 is a master regulator of the wound healing process by promoting the switch between the inflammation and the proliferative phase [44]. The TGF-B activity counterbalances the effect of TNF-alpha in macrophages [45] and favours angiogenesis, ECM deposition and fibroblast proliferation. Hyperglycemia and hypoxia did not have any effect on its gene expression. Hence, hyperglycemia only negatively impacts the expression of pro-inflammatory cytokines but not those involved in wound healing.

## Conclusion

Hyperglycemia has a negative impact on the wound healing of foot diabetic ulcers. High glucose level acts in synergy with hypoxia to maintain the state of chronic inflammation observed in chronic wounds. Hyperglycemia increases the expression of pro-inflammatory cytokines and chemokines by macrophages and decreases their ability of phagocytosis, required for the resolution of inflammation. By contrast, the cytokines involved in wound healing were not impacted by the high glucose concentration. This overview of the macrophage behavior cultivated in hyperglycemia and hypoxia could be helpful towards discovering novel relevant targets for the treatment of foot diabetic ulcers.

## Supporting information

S1 Table List of primer used for the RT-PCR. (PDF)
