# Supplementary material for: Hyperglycemia acts in synergy with hypoxia to maintain the pro-inflammatory phenotype of macrophages
Source: PLoS One. 2019 Aug 15;14(8):e0220577. doi: 10.1371/journal.pone.0220577 (PMC6695165; doi:10.1371/journal.pone.0220577)
Supplement: S1 Table — (PDF) [file pone.0220577.s001.pdf]

**Name**	**primer**
TNF-α Forward	GCCCTGTGAGGAGGACGAACA
TNF-α Reverse	GGTGTCTGAAGGAGGGGGTAATAAA
GAPDH Forward	CAGAGTTAAAAGCAGCCCTGGT
GAPDH Reverse	GAAGGTGAAGGTCGGAGTCAAC
Class B scavenger receptor - Forward	CTGGGCTCTTCACGGTGTTC
Class B scavenger receptor - Reverse	ACTGATCGGAATGCCAGAAGTCAAC
CD36 - Forward	ATTGGGAAAGTCACTGCGACAT
CD36 - Reverse	CGTCGGATTCAAATACAGCATAGAT
SOCS3-Forward	ATCCTGGTGACAT GCTCCTC
SOCS3-Reverse	CAAATGTTGCTTCCCCCTTA
IL-1a Forward	AATGACGCCCTCAATCAAAG
IL-1a Reverse	TGGGTATCTCAGGCATCTCC
IL-6 Forward	GATGAGTACAAAAGTCCTGATCCA
IL-6 Reverse	CTGCAGCCACTGGTTCTGT
GM-CSF Forward	CCTGTGCAACCCAGATTATCACC
GM-CSF reverse	CCCACCCCTTGGTCCCTC
TGF-β1 Forward	GTGGACATCAACGGGTTCACTA
TGF-β1 Reverse	CTCCGTGGAGCTGAAGCAATA
